# Co-producing knowledge on the use of urban natural space: Participatory system dynamics modelling to understand a complex urban system

**DOI:** 10.1016/j.jenvman.2024.120110

**Published:** 2024-02-27

**Authors:** Irene Pluchinotta, Ke Zhou, Gemma Moore, Giuseppe Salvia, Kristine Belesova, Nahid Mohajeri, Joanna Hale, Michael Davies, Nici Zimmermann

**Affiliations:** a Institute for Environmental Design and Engineering, The Bartlett Faculty of the Built Environment, University College London, London, United Kingdom; b London School of Hygiene and Tropical Medicine, London, United Kingdom; c UCL Centre for Behaviour Change, University College London, London, United Kingdom

**Keywords:** Transdisciplinary, Stakeholders, Sustainability, Participatory system dynamics, Participatory modelling, Simulation, Social learning, Thamesmead

## Abstract

Decision-makers are increasingly asked to act differently in how they respond to complex urban challenges, recognising the value in bringing together and integrating cross-disciplinary, cross-sectoral knowledge to generate effective solutions. Participatory modelling allows to bring stakeholders together, enhance knowledge and understanding of a system, and identify the impacts of interventions to a given problem. This paper uses an interdisciplinary and systems approach to investigate a complex urban problem, using a participatory System Dynamics modelling process as an approach to facilitate learning and co-produce knowledge on the factors influencing the use of urban natural space. Stakeholders used a Systems Dynamics model and interface, as a tool to collectively identify pathways for improving the use of space and simulating their impacts. Under the lens of knowledge co-production, the paper reflects how such mechanisms can lead to the co-production of knowledge and social learning. The findings also contribute to identify ways of increasing the value of urban natural space focusing on urban areas undergoing physical and social transformation, such as the Thamesmead case study, London, UK.

## Highlights

• Participatory System Dynamics modelling to co-produce knowledge with stakeholders.
• Using a holistic approach to describe the dynamics of the use of urban natural space.
• The model simulates different impacts on the use of space to inform decision-making.

## 1 Introduction

Understanding and tackling complex urban problems, such as housing affordability, access to services and amenities, and growing health inequalities, requires changes in how we produce and use knowledge. Opportunities for interactions between scientists, decision- and policy-makers, and other actors must be increased to ‘open up the knowledge system’ (Cornell et al., 2013). Notably, there has been an increased interest in widening the nature and form of scientific knowledge production to include a much broader and diverse set of voices to address societal and environmental problems (Stokols et al., 2013), with an emphasis on participatory approaches within environmental decision-making, leading to new forms and types of knowledge production (e.g., Reed, 2008; Reed et al., 2014). A report by Natural England (2022) emphases that engagement is key for more sustainable outcomes and effective decision-making. This study echoes other scholars who have argued that the knowledge used to inform decisions in the urban environment should come from a range of sources: research evidence, practice and the knowledges of locally affected communities (Corburn, 2005; Lawrence, 2020; Wynne, 2004).

The rationale for taking more participatory approaches to decision-making and policy development have been documented across a range of fields, and include: (i) deeper understanding of the issues being explored (e.g., Coletta et al., 2021; Pluchinotta et al., 2018); (ii) engendering trust between different groups (Stringer et al., 2006); (iii) enhancement of participants' knowledge (Wynne, 2004), promoting individual and group learning (Tuler et al., 2017); (iv) improved quality and social legitimacy of decisions and outcomes (Bailey et al., 1999; Holder, 2004); (v) the process of involvement can help build capacity of those involved (i.e. skills, tools, networks) (Kagan, 2006); (vi) Formulate effective policy solutions (e.g., Corburn, 2005; Pluchinotta et al., 2020; Pluchinotta et al., 2019); (vii) improving the legitimacy of strategies (Aminpour et al., 2021). Therefore, an effective decision-making requires knowledge generation. Knowledge is a product of specific values, cultures and relations (Argyris and Schon, 1974) thus it can be helpful to consider the role of different members or groups in the production, or co-production, of knowledge. As Schwermer et al. (2021) note, understanding socio-ecological knowledge diversity across individuals and groups can provide insights into why collective action towards particular goals may not work in practice. In their work on cod-fishing, Schwermer et al. (2021) explored disparities in stakeholders’ knowledges and perceptions in: meanings attached to system components; causal relationships; and, functional implications of perceived changes. They conclude that such disparities are likely to form the basis of disagreement in fisheries management, thus it is important to capture and represent different knowledges and perceptions of divergences. Creating spaces for the involvement of range of stakeholders, and the co-production of knowledge, can build a holistic, integrated knowledge system.

Increasingly in environmental research, the role of participatory and engagement-focused approaches that lead to the ‘co-production of knowledge’, are being used in an attempt to bring together and integrate cross-disciplinary knowledge to generate effective solutions, and transformative outcomes (Moore et al., 2022; Pineo et al., 2021). There are different definitions of knowledge co-production, our work adds to Audia et al. (2021) understanding of co-production as “an interactive and complex process in which disciplines, practices, and knowledge systems can confront, shape, and be shaped by each other, whether by conflict or by cooperation”. Practically, co-production of knowledge can involve inclusive and flexible processes to integrate diverse perspectives, elicit understandings, co-produce new knowledge and encourage an active role in decision-making with (and within) different actors (e.g., Aminpour et al., 2021; Pluchinotta et al., 2022).

Simulation modelling-based learnings are increasingly being used to support stakeholders’ environmental decision-making (Sterman et al., 2013). While the model can sometimes be built without stakeholders, participatory modelling, with its various types of methods, has emerged as a powerful tool that can (i) enhance stakeholders knowledge and understanding of a system and its dynamics under various conditions, as part of a process of social learning, and (ii) identify and clarify the impacts of solutions to a given problem, usually related to supporting decision making, policy, or management (Kelly et al., 2013; Voinov and Bousquet, 2010; Voinov et al., 2016). These benefits have resulted in the adoption of participatory modelling in a range of context, including transdisciplinary research on societal problems, for instance within the Complex Urban Systems for Sustainability and Health (CUSSH) project. Within CUSSH a form of participatory modelling was used to harness the benefits of sustainability-oriented policies, while minimizing the potential adverse consequences of global technological, environmental and social change (Davies et al., 2021; Moore et al., 2021). The participatory process encompasses various forms of collaboration among practitioners, academics, and other stakeholders who engage in a purposeful learning process that elicits and formalizes the implicit and explicit knowledge of participants to support decision-making and action (Sterling et al., 2019).

Within this context, this paper aims to use a participatory System Dynamics (SD) modelling process and the creation of a simulation model as an approach to build learning and co-produce novel knowledge on the use of natural space (henceforth called Use of Space, UoS), whilst taking a holistic approach to investigate the factors influencing the UoS.

Within England, specifically, there has been a decline in the use of natural space in the past decades (e.g., Boyd et al., 2018; Natural England, 2019). Studies suggest that the decline in use is the result of a number of factors, including socio-demographic change, population health conditions, as well as the quality and proximity of the natural spaces and their facilities (Kruizse et al., 2019; Stessens et al., 2021; Tzoulas and James, 2010; Zhang et al., 2020). These influencing factors are often addressed with in isolation, whereas their interdependencies possibly determine why, when and how natural space is used (Salvia et al., 2022). The UoS can be interpreted and analysed as a complex system characterized by interdependencies. Morecroft et al. (1995) explicitly discussed the unintended consequences of overlooking interdependencies. According to the SD literature, using a holistic approach to understand the relationships and interdependencies between the influencing factors is essential to diagnose the root causes, understand co-benefits and develop appropriate and effective solutions (Sterman, 2006).

Our paper builds on theoretical and conceptual approaches from SD, simulation methodologies, socio-environmental participatory modelling, and engaged practice. The starting point for this work rests with a participatory SD modelling approach (e.g., Andersen et al., 1997; Krueger et al., 2012; Scott et al., 2016; Stave, 2002; Videira et al., 2010), to explore an urban environmental issue, identified by project stakeholders (i.e., the use of urban natural space). Following the participatory modelling practice, our work included stakeholders in model building and scenarios testing. Stakeholders often hold valuable knowledge about socio-environmental dynamics and collaborative forms of modelling produce important ‘boundary objects’ used to collectively reason about environmental problems (Gray et al., 2018). In this context, a boundary object is a shared representation of dependencies that participants can modify, that builds trust and agreement (Black and Andersen, 2012). The shift towards more collaborative and engaged approaches to modelling, applying mechanisms that enable, what Flanagan et al. (2021) call the ‘crowding in’ the necessary knowledge, could, in the context of UoS, lead to more diverse, higher quality spaces, and ultimately increase the usage of such spaces. However, the participatory process may be far from straightforward: including several sources of knowledge and understanding different decision-makers’ perceptions can be difficult. Often, indeed, users' language and conceptualisation of natural spaces may be different to that of the professionals who have designed or are managing the spaces. As a result, there is a need to apply and refine participatory mechanisms to co-produce knowledge. This study applies participatory SD modelling to the topic of urban natural space, and the use, of it, to co-produce knowledge.

This paper summarizes the participatory modelling process carried out to co-produce knowledge with local stakeholders within a regeneration initiative based in Thamesmead, London, United Kingdom. It presents the SD model describing the dynamics of the UoS, including a large set of variables (tangible and intangible) and their relationships (linear and nonlinear) identified by a diverse range of stakeholders. The model effectively captures endogenous dynamics between the UoS and wider range of system elements (e.g., maintenance capability, perception of safety, community participation) as elicited from the case study stakeholders during the modelling process. The model and the co-produced knowledge are then used by the stakeholders to collectively identify, through an interface, possible strategic pathways for improving the UoS and simulating the impacts on the system. The findings aim to identify ways of increasing the value and co-benefits of urban natural space through a focus on increasing use, particularly in urban areas undergoing a regeneration process with significant physical and social transformation. Under the lens of knowledge co-production, the paper reflects if and how such participatory mechanisms can lead to mutual, social learning (i.e., the process of learning from each other).

The paper is organised as follows: Section 2 explores the UoS through participatory SD modelling and knowledge co-production. After a brief description of the SD modelling process and of the case study (Section 3), Section 4 presents an overview of the resulting model. Section 5 discusses the strategies impacting the UoS tested and simulated by the case study stakeholders. Section 6 reflects on the contribution of the model under the lens of co-production, concluding the paper.

## 2 Knowledge co-production through participatory system dynamics modelling applied to the use of urban natural space

### 2.1 Overview: the use of natural space and its effects for health and wellbeing

Natural space can be considered a generic term covering a range of spaces, in size, form and function, both formal and informal, which is accessible or open to the public (in contrast to privately owned spaces). Such natural spaces, in urban areas, include parks, canals, riverways, play-areas, cemeteries, public squares, city farms and urban greens (to name a few). There is a substantial body of empirical research on the impacts of natural space on the urban system, with multiple benefits ranging from regulating climate, supporting biodiversity, natural flood management strategies, provision of amenity spaces, social cohesion, and even providing economic value in some areas (Gascon et al., 2016, 2017; Molla, 2015). Increasingly, there is a focus, both within research and practice, on the value of urban natural spaces human and planetary health and wellbeing. The benefits of urban natural space, for health and wellbeing are wide ranging, including: encouraging physical activity and exercise, e.g., lowering instances of cardiovascular health issues (Hartig et al., 2014); improving cognitive function and social development in children; reducing social isolation; and improving mental health at a population level, e.g., reducing anxiety (Lovell et al., 2020). More frequent exposure to urban natural space has been associated with increased life satisfaction and lower inequalities in health in some urban communities (WHO, 2017). The wellbeing dimension of urban green spaces are discussed in detail in Pinto et al. (2022). Veen et al. (2020) developed a model to outline the benefits of urban natural space, at both an individual and community level, introducing the trinity of benefits from either being active or present in the space: physical health, social cohesion, psychological wellbeing.

Natural space can impact individual and community health in different ways therefore different influencing factors (e.g., designs, policies, engagement programmes, social changes), may affect various pathways and health outcomes (e.g., physical, social, psychological). For example, during the COVID-19 pandemic there was an increase in use of natural spaces as places for exercise, relaxation and as an important place for social connection within urban areas (ONS, 2021). The UK Government ‘People and Nature Survey July 2020’ (Natural England, 2020) found that 46% respondents reported spending more time outside than before COVID-19, and that urban green spaces were the most common type of space used, with 50% of adults surveyed visiting urban parks.

Planned interventions aiming to increase the UoS often focused on targeting individual and tangible issues, such as investing in the maintenance and cleanliness of spaces (Greater London Authority, 2009). While such focus helps to ensure natural spaces are clean and well maintained, these mono-focused interventions do not address broader issues (for instance, accessibility, safety or inequality of use amongst certain population groups) that may be influencing use, and neglect to understand the drivers and motivation behind UoS for different users. There are limited advantages in focusing on particular issues, such as access (e.g., Sharifi et al., 2021), design and multi-functionality (e.g., Belmeziti et al., 2018) rather than considering the influencing factors together, holistically. There is a need to understand how the different factors interact and influence usage – as a system. Thus, there is value in bringing together research from a range of disciplinary perspectives to consider UoS. Research fields, such as urban ecology, encourage us to consider the connections among ecosystem services, human wellbeing, and the interactions within environmental systems (Grimm et al., 2008), and methodological approaches, such as participatory modelling and SD simulation models, have a proven track record for tackling messy socio-environmental problems and inform strategy and policy development (Tsolakis and Anthopoulos, 2015). The field of urban ecology continues to evolve, indeed, embracing diverse perspectives and approaches that increasingly concentrate on both the “natural” elements and socio-economic components of entire ecosystems (Wu, 2014).

### 2.2 Co-production of knowledge through participatory system dynamics modelling

Participatory SD modelling and Group Model Building (for an introduction see Andersen and Richardson, 1997; Andersen et al., 1997; Richardson and Andersen, 1995) have gained ground in several fields and with different applications. They are well-known tools for understanding how stakeholders understand the variables, relationships, and feedback loops that comprise a complex system to co-create qualitative causal maps and simulation models (Hovmand, 2014). Researchers (e.g., Thompson et al., 2016) extensively discuss that SD modelling allows to integrate observations, theory and knowledge from multiple perspectives to improve understanding of the structure of a problem, with its causal connections and dynamic hypothesis. Other system dynamicists have long recognized the importance of involving stakeholders in the process of model building (see Lane, 2010; Meadows et al., 1982; Roberts, 1978; Vennix et al., 1990). Mental model refinement and alignment has previously been identified as an outcome of Group Model Building (Dwyer and Stave, 2011; Rouwette et al., 2009; Rouwette and Vennix, 2006; Scott et al., 2013). Modelling undertaken in an open and participatory way is, therefore, considered as a process of building mutual understanding, defining terms and notions, and sharing experiences (Voinov and Bousquet, 2010). A collaborative SD modelling process can develop stakeholders’ understanding of future options and enable a structured exploration of interdependencies (Pluchinotta et al., 2021). Using participatory SD modelling, allows including different perspectives, mental models and understandings (e.g., Ford and Sterman, 1998; Giordano et al., 2017; Pluchinotta et al., 2022; Sterman, 2001), providing an opportunity to tap into the variety of conceptualisations of an issue, such as UoS.

Engaging a range of viewpoints and bringing together a range of actors (including users, designers, planners, and local authority officers) is a key step in co-producing effective solutions or interventions (e.g., Pluchinotta et al., 2019). However, within the SD literature, participation as an explicit means for knowledge co-production is often under-reported, focusing mainly on knowledge elicitation for modelling purposes. For instance, there is only a limited number of research explicitly discussing knowledge co-production, usually investigating the improvement of stakeholders' mental models during participatory processes (e.g., Ford and Sterman, 1998; Hovmand et al., 2012; Kim, 2009; Singer et al., 2017). Luna-Reyes et al. (2008) discuss the capacity for knowledge sharing across organizational boundaries, by modelling the relationships among activities that create shared knowledge among stakeholders. Community based SD modelling prioritizes building stakeholders’ capabilities in the use of tools from the field of system dynamics (Ballard et al., 2020; Hovmand, 2014; Langellier et al., 2019). Whereas Mediated Modelling by Van Den Belt (2004) focuses on bringing together diverse interests to raise the shared level of understanding and foster a broad and deep consensus for supporting decisions.

If diverse stakeholders are going to take part in the participatory modelling process, they need to understand the problem, that the modelling, and ultimately the decision concerns (Sterling et al., 2019): here is where the learning arises. Bakhanova et al. (2020) highlight that talking about learning, researchers either explicitly or indirectly mean social learning because participatory modelling implies a better understanding of the problem through reflecting on a person's own perceptions and considering the perspectives of others. Therefore, stakeholder learning is a fundamental component of participatory modelling processes, both for reasons of knowledge integration and democracy (Hedelin et al., 2021). Indeed, Jordan et al. (2018, p.2) define participatory modelling as “a purposeful learning process for action that engages the implicit and explicit knowledge of stakeholders to create formalized and shared representations of reality”. The reported benefit of social learning is of particular interest to this study, notably understanding if social learning is facilitated through specific engagement processes, like participatory SD modelling. SD modelling processes can aid the formation of knowledge as a dynamic process and a mutual exchange of experience between participants (Zimmermann, 2017). Tuler et al. (2017) also emphasize learning through the dynamic process of developing the capacity to think critically, creatively, and collaboratively in support of individual and group decision-making and planning to remedy a problem of common concern. Henly-Shepard et al. (2015) present a three-phase social learning framework which occurred through iterative modelling to identify the anticipated impacts of a tsunami. They found individual reflection demonstrated single-loop learning, group discussion demonstrated double- and triple-loop learning around: reasons for the varying success of different strategies at achieving the desired results; and revision of the disaster action plan based on the new awareness, understanding and knowledge generated.

Undertaking participatory processes in SD does not guarantee the co-production of knowledge or social learning. As Hedelin et al. (2021) reminds us “process features can become obstacles for knowledge integration, or they can enrich the process” through supporting a better understanding of the complex issue being explored. The challenges of participation have been recognized, even by scholars who advocate the importance of collaboration and engagement. Poor or inappropriate participation can lead to involvement fatigue, tension, conflict, and reduced trust amongst those involved (Stringer et al., 2006). Time and resources need to be devoted to understanding each other, to build ‘relational expertise’ (Edwards, 2021). Unlike just communication, for collaboration and participation increased time and effort may be required to understand different concepts, experiences and values, and develop relationships. Undertaking participation to co-produce knowledge can therefore affect the logistics of project delivery, increasing time frames and resources. Putting aside the logistics of time frames and resources, Lynch (2006), notes that there has to be a willingness from stakeholder to work openly, with a degree of mutual respect, ability to listen, and commitment to work together. Furthermore, it can be challenging to bring together diverse knowledge (e.g., Aminpour & et al., 2021; Schwermer et al., 2021), and reach consensus, on topics or issues where there are multiple conceptualisations (like sustainability, health and wellbeing) (Moore et al., 2022). Scholars, such as Pineo et al. (2021) have called for further reflection and integration on the participatory processes, within the field of transdisciplinary research, to unpack ‘the complex, costly, time-consuming’, yet potentially rewarding’ aspects of participation.

To summarize, through a participatory SD modelling process used as an approach to facilitate learning and co-produce new knowledge, a simulation model on the UoS in Thamesmead (London) was built with a range of local stakeholders. The next sections describe the process we applied, the model, and what it achieved under the lens of co-production.

## 3 Participatory system dynamics modelling in thamesmead

### 3.1 Case study

Thamesmead is a neighbourhood in South-East London (UK), characterised by an extended and interlinked network of urban natural spaces (both, blue and green) with several sites of nature conservation interest: 150 ha of parks and 32 ha of water, with five lakes, 7 km of canals, 5 km of Thames River waterfront. Thamesmead is considered as one of Britain's most ambitious post-war housing projects and it consists of concrete social-housing stocks built from an inhospitable marshland in mid-1960s. About 16,000 households reside in the area, with twice the amount of green space per person than the London average (Peabody, 2019). The first masterplan was never fully realized, and from 2018 Thamesmead is the focus of a 25-year regeneration plan managed by Peabody Housing Association. Broadly, the research activities carried out within the case study aimed: (i) to use a highly participatory SD modelling process to inform decision-making around urban natural space; and (ii) to collectively identify pathways of improvement for the UoS in Thamesmead, thanks to the analysis of systemic interventions at strategic level.

The group of stakeholders involved in the participatory SD modelling process consisted of a Local Government, the Greater London Authority (GLA), Environmental NGOs, the Environment Agency (EA), a Housing Association acting as developers, and a various number of experts from academia in the context of health, sustainability, built environment and co-design. Stakeholders were identified using a snowballing approach (Prell et al., 2008; Reed et al., 2009). Preliminary scoping interviews with project partners were carried out to start up the participatory modelling process. The snowballing sampling was interrupted when participants no longer suggested new stakeholders. For modelling purposes, we needed to consider stakeholders’ specific roles and expertise (e.g., relevant stakeholder working on maintenance of natural spaces); therefore, the stakeholders involved in the modelling process were selected according to their role and competences. All the key organizations, involved in the regeneration project, were represented, while representatives of residents were involved in different phases, e.g., for the qualitative modelling process to include their perspective into the model (as discussed in detail in Salvia et al., 2022), and to investigate possible actions to improve the UoS.

This paper focusses only on the quantitative participatory modelling process (described in Section 3.2) of a wider case study within two large-scale projects, i.e., Complex Urban Systems for Sustainability and Health^1^ (CUSSH) and Community Water Management for a Liveable London^2^ (CAMELLIA). It is worth underlining that the case study stakeholders jointly agreed on the model focus (namely, UoS), during engagement activities prior to the ones described in this paper. The qualitative modelling phase is described in Pluchinotta et al. (2022); it allowed the stakeholders to identify their priorities and collaboratively discuss the group's preferred model focus.

Therefore, the SD simulation model (described in Section 4 and Appendix II) captures the factors influencing of the UoS and their interdependencies, as identified by the case study stakeholders; namely, usability and accessibility, maintenance and space condition, residents' perceived safety and awareness of the spaces, community participation, biodiversity, time constraints and structural poverty. Through an accessible interface, the model allowed the stakeholders to test different scenarios together exploring the impact on space use by capturing the dynamics between the influencing system elements, co-producing knowledge.

### 3.2 The quantitative system dynamics modelling process and participatory engagement

The UoS simulation model is based on a Causal Loop Diagram (CLD) focusing on the space use, distilled from four wider CLDs developed over a number of participatory workshops with the Thamesmead stakeholders (described in Pluchinotta et al., 2022). Afterwards, the modelling team used literature, experts' opinions, and stakeholders' knowledge to quantify and validate the model. The participatory quantification process, highly grounded on the stakeholders’ knowledge is described in Pluchinotta et al. (2024). The model was then used by the stakeholders to collaboratively test scenarios and strategies.

For the sake of brevity, the modelling steps used to co-develop with the stakeholders the SD simulation model on the UoS are detailed in Table 1. For each step, objectives, methods, outputs are reported. Total number of participants during each phase is reported in the last column of the table, together with the type of stakeholder group. The specific source of information for each variable is outlined in Appendix III. This paper directly links the modelling process to the case study activities; however, the modelling steps are generalizable and replicable.

**Table 1:** Steps of the Participatory System Dynamics (SD) modelling process. CLD = Causal Loop Diagram.

Phase	Step	Objective(s)	Method(s)	Output(s)	No. of Participants	(No.) and type of Participant
Qualitative modelling phase: Focussed CLD building	Building an aggregated CLD on the use of space	- To synthesise the four different CLDs focusing on the quality of public spaces built during the participatory qualitative modelling phase	- One of the SD modellers aggregated the variables and relationships between variables from a set of CLDs elicited during previous workshops. - Four rounds of discussions between the SD modellers	- Aggregated and focussed CLD used for building the simulation model	3	(3) SD modellers
Quantitative modelling: System Dynamics model building	Building the use of space simulation model	- To build the structure of the simulation model from the aggregated CLD - To identify gaps in model assumptions	- Several individual and group working sessions between the SD modellers to identify stocks and flows from the aggregated CLD	- Simulation structure of the use of space SD model - Identification of the list of variables, relationships, and sectors for quantification	2	(2) SD modellers
Quantitative modelling: quantification and model validation	Quantification and parametrization of the use of space SD model	- To quantify model relationships and input variables - To calibrate model using empirical data and parameterise variables using different source of information	- Scoping literature review of specific model sections - Online questionnaire with academia experts of several domains e.g., related to sustainability, health, co-design and urban regeneration to gather quantification data and to weight different variables - Information processing and internal working sessions - Two ad-hoc workshops with relevant local stakeholders (online, 1 h)	- Baseline value for variables elicited by participants and experts - Quantification of relationships across simulation model sectors elicited by participants and experts - Calibration tests - Identification of modelling gaps that need further information	11	(3) SD modellers (4) Experts from academia (4) Housing association
	Model presentation and structure validation	- To present and validate the structure of the SD model - To support the quantification of the model	Stakeholder workshop for the presentation of the model, followed by guided group discussions for validation and Behaviour Over Time (BOT) graphs building to generate the initial values of key parameters and reference modes (online, 2 h)	- Validated structure of the UoS model by participants - BOT graphs used for supporting the model quantification by participants	13	(2) Local Government (2) Environmental NGOs (2) Housing Association (1) Architect involved in one of the regeneration projects of the area (4) Facilitators/modellers (2) Observers from academia
	Reference model validation	- To identify the reference model for the UoS SD model	Stakeholder workshop to discuss the historical datasets available to be used for model calibration (online, 1 h)	- Suitable reference model suggested by participants	16	(4) Local Government (1) Environmental Agency (1) Environmental NGOs (3) Housing Association (7) Facilitators/modellers
Quantitative modelling: Scenarios building and analysis	Model finalisation and interface building	- To complete the model building by integrating validation information and modelling testing - To build the model interface	- Calibration and sensitivity analysis - Internal working sessions for interface building	- Final model - Interface	3	(3) SD modellers
	Scenarios building in interface	- To generate a set of policy scenarios - To include the scenarios in the model interface	- Set of 4 stakeholder workshops to co-design a set of policy alternatives −2 internal working sessions to combine thepolicy alternatives in modelling scenarios, using an adapted version of the approach described in (Pluchinotta et al., 2019) (online, 1–2 h)	- Integration of policy alternatives in the interface - Co-designed set of scenarios to test by participants and SD modellers	11	(3) Facilitators/modellers (6) Residents (2) Housing Association
	Scenario analysis	- To analyse the model behaviour using the designed scenarios generated by participants	- Internal working sessions between modellers (online, 1–2 h)	- A list of scenarios and strategies - Scenarios analysis	2	(2) SD modellers
Quantitative modelling: Strategy development and learning	Strategy development and learning	-To present the final SD simulation model - To allow the stakeholders to explore and interact with the model via the interface - To discuss the key elements influencing the use of space to inform decision- making - To discuss how to use the model and plan the next steps	- Set of 2 stakeholder workshops for presenting the final interface, interacting with the model, and discussing the findings from the scenario analysis (online, 3 h)	- Stakeholders' use of the interface in workshops to support discussions and learning - Stakeholders' identification of strategies to improve the use of space in Thamesmead	16	(3) Local Government (1) Environmental Agency (2) Water utility company (5) Housing Association (5) Facilitators/modellers

Finally, we note this study started before the COVID-19 pandemic, however the key modelling activities described in this paper were undertaken during and after the national lockdowns. With regards to the processes and mechanisms of participation adopted within this study, there was a shift from the delivery of face-to-face workshop to online workshops (discussed in Zimmermann et al., 2020). A key drawback was the difficulty in building rapport and noticing non-verbal cues (Lo Iacono, et al., 2016).

## 4 Model overview and behaviour

### 4.1 The Causal Loop Diagram of the simulation model

**Fig. 1:**
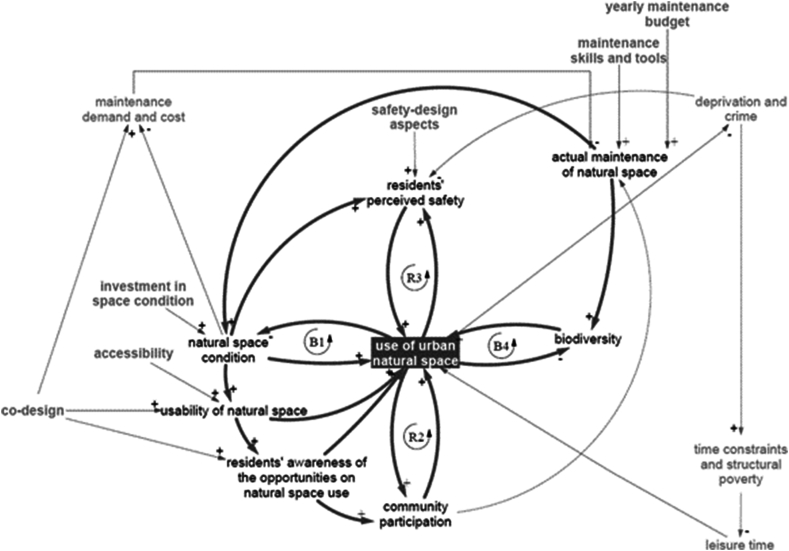
Simplified Causal Loop Diagram (CLD) representing the key dynamics behind the Use of Space (UoS) simulation model as identified by the stakeholders during the participatory modelling process. R: Reinforcing Loop, B: Balancing Loop.

The following CLD (Fig. 1) represents the simplified causal model behind the simulation model and it captures the key factors and interdependencies influencing the UoS, according to the stakeholders involved in the participatory modelling process.

According to the case study stakeholders, the UoS is influenced by the ‘natural space condition’: the better the space condition the more it will be used, but on the other side, the UoS will speed up the decay process reducing the condition of the natural space (balancing loop B1). Investment in space conditions and maintenance activities can improve the space condition. Within the model, if there is not investment programme there is no improvement of the space condition.

The ‘usability of the natural space’ is influenced by their condition, and it influences the use. ‘Accessibility’ influences the usability too. The usability variable describes the space's physical elements that directly influence the possible UoS, while the accessibility is the ease with which parks can be reached and used from one location (Park, 2017) (for instance a combination of the following elements: physical obstacles and challenging topography of the paths networks, new public transport opportunities, availability of public/private transports, access standards, facilities, and amenities (Public Health England, 2020)). Similarly, the higher the usability, the more residents are aware of the opportunities related to the use of Thamesmead natural space. This awareness directly influences the use. Furthermore, an increased residents' awareness of the opportunities implies the increase of community participation. A second loop (reinforcing loop R2) shows that the increase of ‘community participation’ boosts the UoS, and the more the spaces are used, the more community members feel like participating. Within this context, ‘community participation’ is defined as the fraction of residents participating in the maintenance of the local natural areas. Within the model, ‘community participation’ is based on the percentage of residents that are aware of local opportunities to use local natural space with a delay of time needed until some of them start participating (allowing the awareness to be translated into behaviour); this is also influenced by the time residents have available to participate engagement events, and their actual UoS.

In relation to the condition of the space, according to the case study participants, the condition influences the residents' perceived safety. The third loop (reinforcing loop R3) highlights that if the space is perceived safe, more residents will use that space, and an increase of use causes and increase of the perceived safety, too. On the one hand, within the model, ‘residents’ perceived safety’ can be negatively influenced by ‘deprivation and crime’. On the other hand, space condition, UoS, safety-design aspects (such as increasing surveillance and street lighting and decreasing dead ends) can increase the perception of safety.

Looking at the sustainability aspect of the UoS, a fourth loop (balancing loop B4) underlines that, according to the stakeholders, the more the place is used, the less biodiversity survives, and on the other side more biodiversity increases the attractiveness of the natural spaces, increasing the use. Introducing the role of maintenance, according to the stakeholders, maintenance improves the space condition, slowing down the decay, and it is constrained by the maintenance skill, tools and budget. Biodiversity can improve thanks to the maintenance of the natural space, and community participation can increase the maintenance capacity and the actual maintenance itself if community members engage in maintenance activities. Generally, the higher the use of the space, the higher the maintenance demand. Within the model, the maintenance capacity represents not only the budget that the main owner of the Thamesmead public space planned, but also the skills and tools the maintenance team holds. The maintenance costs are put under pressure by a decrease of the space condition caused by the UoS. Under-maintained natural space will ultimately reduce its use.

According to the case study stakeholders, the engagement of local residents through the use of co-design process has a greater impact on their use, namely it is more likely that the spaces are fit for purpose, explaining why the use of co-design approaches increases also the usability of the space and the awareness of the opportunities. Yet, the use of ‘co-design approaches’ may also increase the ‘maintenance demand and cost’ because community participation may lead to more maintenance-intensive design of the space. Lastly, deprivation and crime not only reduce the perceived safety, it also increases residents' time constraints and structural poverty. In case of reduced availability of leisure time, the spaces will be used less. In our simulation model, leisure time is also influenced by the presence of local job opportunities and services, as the lack of local services was mentioned as an important reason but probably not the only one for the lack of leisure time for attending urban natural spaces. For instance, a complementary study mentions limited availability of local services and amenities (including shops, restaurants, schools and entertainment venues) and of local job opportunities will push the residents to spend more time traveling, thus eroding the time they can spend in local green space (Salvia et al., 2022).

### 4.2 Simulation model assumptions and parameters

**Fig. 2:**
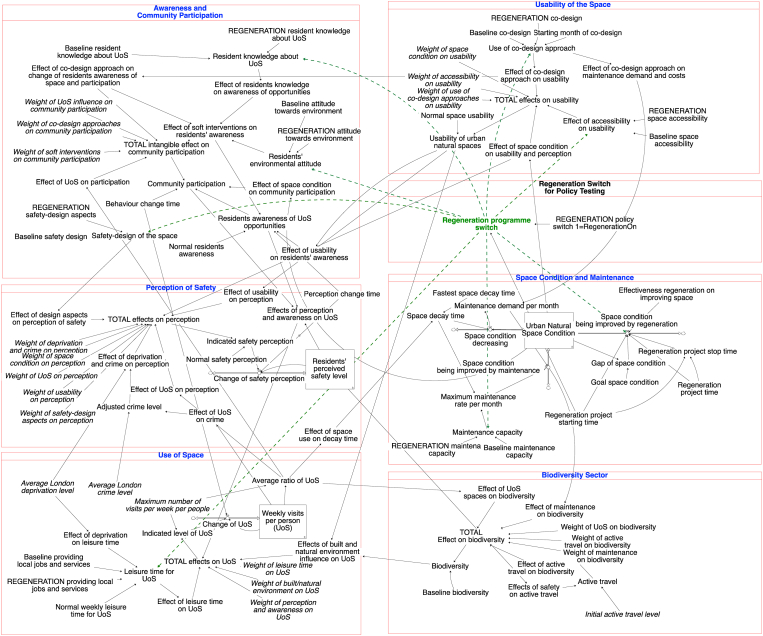
General overview of the Use of urban natural Space (UoS) System Dynamics (SD) simulation model.

This section provides a general overview of the stock and flow model structure (Fig. 2) and underlining assumptions. Appendix II includes the model documentation.^3^ The conceptual representation of the model is showed in Fig. 1. The model includes three stocks: natural space condition, residents' perceived safety level, and the weekly individual use of natural space. The initial value of space condition and residents’ perception of safety of natural spaces were estimated by the stakeholders while discussing the reference modes of key parameters (see Table 1 for details on the modelling process); while the initial value of the weekly UoS per people was calibrated using the Monitor of Engagement with the Natural Environment (MENE) dataset (see Section 4.4). Due to the conceptual and highly aggregated nature of many of the variables, we often used dimensionless indices. For example, space condition is a dimensionless value ranging from 0 to 1. While 0 means the space is not useable at all, a value of 1 represents that it is in perfect condition. The baseline values were estimated by the stakeholders during the participatory modelling sessions.

The model was built using Stella Architect^4^ software and it simulates a 60-year (720 months) time horizon, from 2000 to 2060. The long-term horizon was chosen as following the information gathered from the stakeholders, the model includes a 25-year regeneration programme from 2018 to 2043. It provides sufficient time span to identify longer-term comparisons before and after the end of the programme. The model assumes that the effects of improvements will continue even after the regeneration programme ends (e.g., the effect of usability, safety-design, effects of co-design and other engagement approaches), however the process of improving the natural spaces condition as a direct inflow to the space condition ends with the regeneration programme.

Considering the participatory nature of the modelling process, this model is highly grounded on the causal relationships identified by the case study stakeholders. To quantify the causal relationships, several effect variables are included, such as the effect of accessibility on UoS, effect of space design elements on perception of safety, and effect of co-design on awareness. We assume positive S-shape effects (e.g., effect of usability on residents' awareness, effect of leisure time on use of blue green space) for positive polarity links, and negative S-shape effects (e.g., effect of deprivation on leisure time) for negative polarity links. The S-shapes indicate a decreased marginal influence. This modelling decision was made to facilitate the knowledge co-production and related learning process and to efficiently capture a large set of intangible variables and non-linear relationships. In addition, a set of non-linear relationships and their weights in influencing the output variable are used. The weights show experts' perceptions of the variables. Each variable's influence on the end variable is weighed by experts individually. The final value of weights was averaged among experts. For instance, the effects of safety-design aspects, usability, space condition and use of BG space affect the perception of safety with a different strength.

Due to the lack of research literature on some of the interdependencies captured by the model, the quantification of the effect-relationship variables, non-linear relationships and weights was largely dependent on the participatory modelling process described in Table 1. The specific sources of variables are outlined in Appendix III. The quantification process carried out enhances the model's relevance in supporting the local stakeholders in understanding the interconnectedness of the UoS system as part of a real-world case study. The trade-off of modelling several soft and intangible variables, and nonlinear relationships under data scarcity is the rather conceptual nature of the resulting model and its simulation outputs.

### 4.3 The main model sections

The simulation model includes five sectors. Firstly, in the space condition and maintenance sector, the stock ‘urban natural space condition’ represents the physical condition of the space, which is moderated by an inflow ‘space condition being improved by regeneration’, an inflow of ‘space condition being improved by maintenance’ and an outflow ‘space condition decreasing’. The inflow of regeneration improvement is regulated by the ‘regeneration programme switch’, with a start time and a stop time. If the regeneration programme switch is turned off, there is no improvement of the space condition from regeneration. If the programme switch is turned on, then the space condition adapts to the desired space condition when there is a gap. The outflow equals the stock of space condition divided by the ‘space decay time’. The fastest space decay time used for the model is 7 years, which is influenced by (i) the use of space, a higher use of space will speed up the decay process; and (ii) the actual maintenance, a higher level of maintenance will slow down the decay process. The 7-year time frame for an urban natural space to decay without maintenance until the condition “not suitable for human use” has been identified by the stakeholders and an urban planner expert during the validation workshop and by an urban planner expert.

The second sector focuses on the usability of urban natural space, describing the space's accessibility and design features that directly influence the possible use of the space. The usability can be increased by the use co-design approach (weight = 0.2), accessibility (0.4), and the space condition (0.4). the use of co-design approaches before the regeneration programme is assumed to be 0, and only takes place when the regeneration starts. The use of co-design approaches also influences maintenance demand and cost in the first model sector, as mentioned by the stakeholders during the validation workshop, and by a co-design expert. In contrast, better space condition demands less maintenance, indicating a decrease of maintenance demand and cost.

The third sector regards residents' awareness and community participation. ‘Residents’ awareness of ‘how to use’ of spaces' is a delayed response of 36 months (according to expert judgment), which is assumed to be the perception change time. In System Dynamics modelling, delays refer to situations where changes to the system do not manifest immediately (Sterman, 2000). The delay of residents' awareness suggests the lapse between the initialisation of interventions, such as community participation and safety-design, and the time it takes for residents to become fully aware of the changes in spaces. The ‘residents’ awareness of UoS opportunities' is influenced by the accessibility of the area and soft interventions on raising residents' awareness such as the environmental attitude, and use of co-design approaches. Whereas ‘community participation’ closely connects with residents' perceptions of safety. Specifically, the UoS (weight = 0.2), co-design approach (0.4), and soft interventions in raising residents' awareness (0.4) intangibly impact the community participation, together with the effect of space condition.

The fourth sector addresses residents' perception of safety of the area. The variable ‘residents’ perceived safety level’ is a stock, which tries to reach the ‘indicated safety’ target. The target is influenced by the current level of deprivation and crime, space condition, UoS, usability and safety design aspects of the spaces. Closing the gap between the current safety level and the target requires time. Experts estimated the time to perceive changes in the indicated safety to be three years. Within the model, the ‘indicated safety’ depends on several factors, namely, the safety-design features of the space (such as increasing surveillance, street lighting, and decreasing dead end, etc.) (weight = 0.2), usability (0.1), space condition (0.2), use of space (0.2) and the deprivation level (0.3). We used the London crime index and deprivation decile to parametrize the effect of deprivation and crime on perception. According to the stakeholders' understanding, crime declines if more people use the space.

The last sector focuses on the UoS. We modelled the UoS stock as a ‘weekly visits per person’ to capture the frequencies of UoS (called also ‘average number of weekly visits per person’). The indicated UoS is influenced by the leisure time availability (weight = 0.4), usability of natural space (0.2), safety perception and awareness on UoS opportunities (0.4). Leisure time availability for using the natural space is influenced by the average London deprivation decile, and the availability of local jobs and services. Normal leisure time for using the space is assumed to be 120 min per week per people, after adjusting the influence of baseline job opportunities, and the deprivation decile, the leisure time to use the space is 45 min per week per people.

### 4.4 The reference model and calibration

**Fig. 3:**
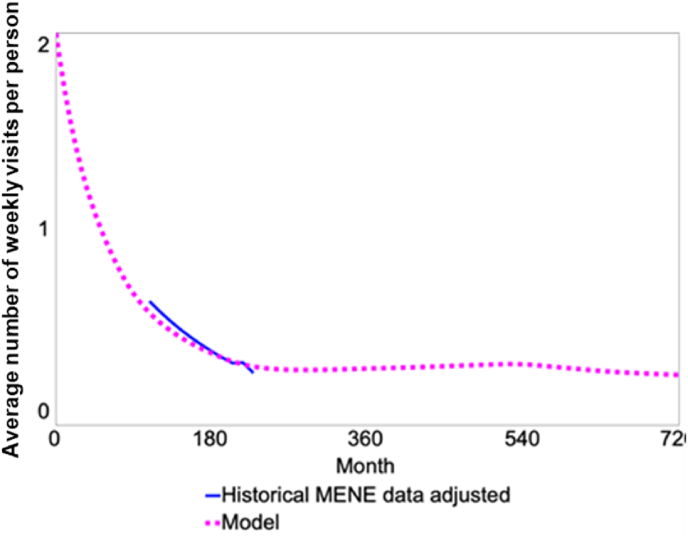
Model calibration, historical dataset compared with the baseline scenario of the model (Month 0: year 2000, Month 216: year 2018, Month 720: year 2060).

We obtained the data on natural environment visits from the Monitor of Engagement with the Natural Environment (MENE) survey conducted from March 2009 till February 2019 by Natural England in partnership with the Department for Environment, Food and Rural Affairs (Defra) of the UK Government (Natural England, 2019). MENE is a UK national dataset based on a survey collecting data on natural environment attendance, and it provided the average number of weekly visits to natural spaces from 2012 to 2019. MENE survey was conducted through home visits, asking respondents to report the number of visits they made to the natural environment over the seven days preceding the day of the survey and recording the date of the visit, first part and the first character of the second part of the postcode where the respondent resides, and respondents' socio-economic characteristics, including respondents’ age, sex, household size, working status, and housing tenure. They survey sampling method draws on a representative sample of the English adult (>16 years) population selected using a random location sampling method described in detail in the technical report of the survey (Natural England, 2019). In order to obtain the reference mode for simulation, which is the real-world mode of behaviour that our model is supposed to replicate, we extracted data on all respondents residing in Thamesmead, as identified by the postcode (DA18, SE28, SE2 9), restricting it to the visits that were made within 5 miles of the residence to reflect the natural environment visits made locally within Thamesmead (as represented in Fig. 3).

As the yearly data included random variations, to examine whether and how natural space visits have changed in Thamesmead over time, we used Poisson regression model of visit rates per person per -week. The models were adjusted for the month of the visit (to control for seasonal effects), respondents' age, sex, household size, working status, housing tenure, and ethnicity to control for any deviations from the whole population that were caused by the sample drawn. After much discussion, we excluded the year 2018 from the analyses, as it seemed to be a clear outlier in the data which, depending on the method for estimating the trend, even changed the trend's direction. In Fig. 3, the blue line represents the historical MENE dataset from 2009 to 2019, showing a decline in the UoS from 0.63 to 0.27 per person per week, while the red line is the baseline scenario of the model without any regeneration intervention.

## 5 Scenarios testing and learning

**Fig. 4:**
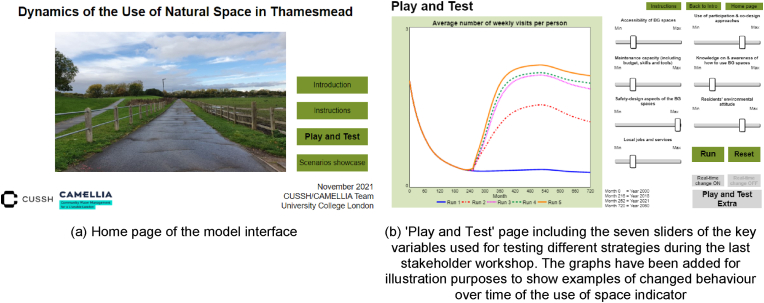
Home page of the model interface (a) and example of the ‘Play and Test’ page (b) used for testing different scenarios and strategies during the stakeholder workshop.

Thanks to the accessible interface (Fig. 4), we used the model as a platform to engage with the stakeholders. We aimed to: explore and discuss possible pathways of interventions for improving the UoS in Thamesmead, and investigate what to prioritize, enabling social learning. The ‘introduction’ page of the interface provided overview of the model mechanisms (see Fig. 4 (a)). The ‘play and test’ page of the interface included seven sliders for the key variables and graphs with the simulation outputs; the stakeholders were able to modify the sliders and to instantaneously see the impact on changed behaviour over time of the key indicator (i.e., average number of weekly visits per person) (see Fig. 4 (b)).

The agenda of the last workshop (‘strategy development and learning’) is detailed in Appendix I. Briefly, during the last workshop, the participants, firstly, individually selected three variables that they considered important for improving the UoS, from the existing list of seven key input model variables (described in Table 2). They then discussed their preliminary thoughts on how influential these variables are. The most voted variables were then discussed and ranked with the followed order: 'Accessibility of the urban natural space' (9 votes), 'Safety design aspects of the urban natural space' (6), 'Use of co-design approaches' (5), 'Knowledge on & awareness of how to use natural space' (5), 'Maintenance capacity' (5), 'Local jobs and services' (proxy of leisure time availability) (2), 'Residents' environmental attitude' (with zero votes).

**Table 2:** Key input model variables of system elements influencing the Use of Space (UoS), modified by the stakeholders through the interface during the strategy development workshop. Each variable is dimensionless.

Key input variables	Definition	Number of votes from the stakeholders during the workshop
Safety design aspects of the urban natural space	Physical characteristics of the space influencing the resident's perceived safety e.g., oriented towards improving street lighting, increasing surveillance, decreasing dead ends, etc.	6
Maintenance capacity (including budget, skills and tools)	It represents the budget available and the skills and tools the maintenance team holds.	5
Accessibility of the urban natural space	It indicates the presence of access standards e.g., in relation to the presence of physical obstacles, challenging topography, availability of public/private transports, etc.	9
Use of co-design approaches	The use of approaches for designing a space attempting to actively involve all stakeholders, to ensure the result is useable and meets their needs, increasing knowledge on/awareness of local opportunities in the space use	5
Residents' environmental attitude	It represents the residents' pro-environmental behaviours. It allows individuals to understand environmental issues and take action to improve the environment.	0
Knowledge on & awareness of how to use natural space	It represents residents' knowledge on how to use the urban natural space and awareness of local opportunities related to space use.	5
Local jobs and services	It represents the element influencing leisure time availability for attending natural space. Deprivation, crime and time constraints caused by long commute, reduce the time availability	2

Afterwards, participants were divided in groups and using the interface they tested different jointly agreed scenarios to see how much the UoS can be increased by modifying a set of three variables per time, naming and comparing each scenario with the baseline and the other model runs. Before each simulation run, stakeholders were asked to anticipate and discuss any behaviour of the interventions. This was an iterative process in which participants were testing out ideas, discussing options, sharing and building knowledge together (between 4 and 7 scenarios were tested by each group). A plenary discussion was then held presenting the developed strategies: this closed the activity.

The workshop was delivered online (via Microsoft Teams) and the session was recorded, transcribed, and analysed. Notetakers helped capturing screenshots of each tested scenario, recording the variables changes and their intensity. The notes included also participants’ comments on the scenarios testing, and the workshop recording helped improving them. An online tool for collaborative work (i.e., Miroboard) was used to record the scenarios tested by the different groups and to support the discussion during the workshop. The screenshot of each scenario tested was pasted on a template prepared by the workshop organizer, allowing to quickly visualize the different scenarios and compare them during the group discussion. This way of recording and recapping the scenarios facilitated the learning process.

**Fig. 5:**
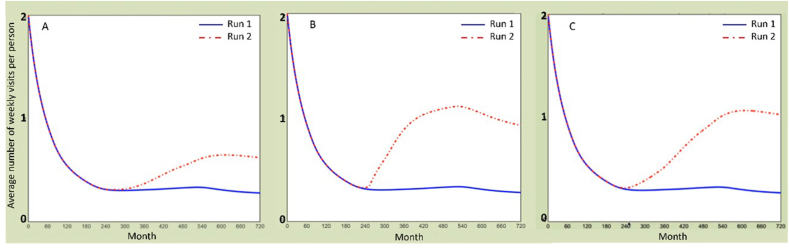
Screenshots of the first scenarios tested by each group of stakeholders during the strategy development workshop. The stakeholders named the scenarios. Y-axis: UoS indicator, i.e., average number of weekly visits per person. X-axis: Months (Month 0: Year, 2000; Month, 2016: Year, 2018; Month 720: Year, 2060). Run 1: baseline scenario, Run 2: first scenario tested by the participants.

**Fig. 6:**
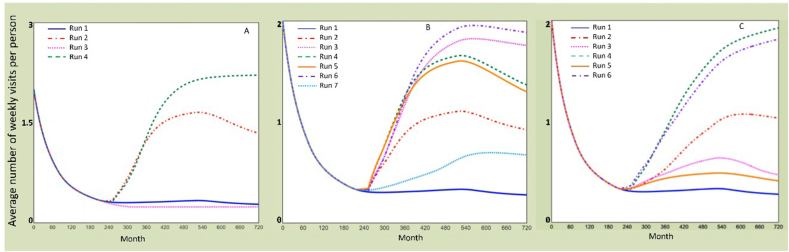
Screenshots of the last scenarios tested by each group of stakeholders during the strategy development workshop. The stakeholders named the scenarios. Y-axis: UoS indicator, i.e., average number of weekly visits per person. X-axis: Months (Month 0: Year, 2000; Month, 2016: Year, 2018; Month 720: Year, 2060). Run 1: baseline scenario. Group A: Run 4 - best and last scenario. Group B: Run 6 – best scenario, run 7 - last scenario. Group C: Run 4 - best scenario, run 6 - last scenario.

The following Fig. 5, Fig. 6 show the screenshot of the first, best and last scenarios tested by each stakeholder group during the workshop; Table 3 summarizes the values of the seven key variables, changed comparing to the baseline (the variables values that were changed are highlighted in grey). A minimum value stands for not existing or very low, the middle one means acceptable, while the maximum value refers to excellent.

**Table 3:**
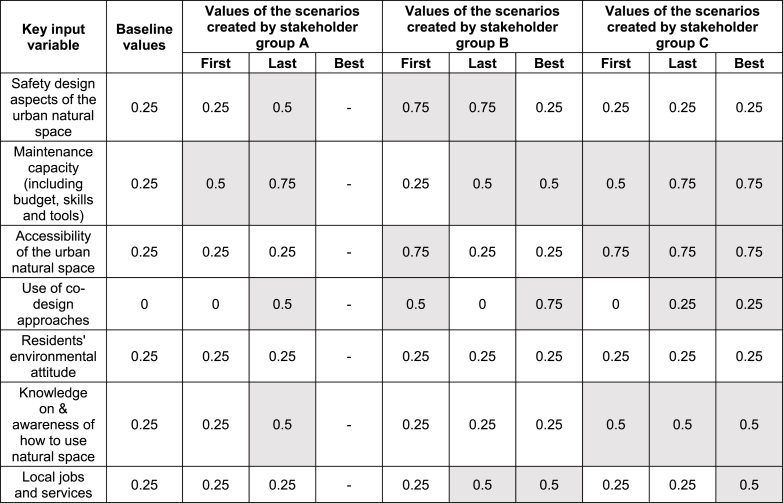
Variables values for the first, last and best scenarios created by each stakeholder groups, compared with the baseline scenario. The variables values that were changed from the baseline values are highlighted in grey.

The ‘best’ scenarios are the one with the highest increase of the UoS indicator, namely the average number of weekly visits per person. The last scenarios were not always the best one tested, however stakeholders preferred to run different combinations before the end of the activity. The evolution of the scenarios underlines how participants gained knowledge as the workshop progressed.

In the following section a summary of the learnings on the UoS, from the scenarios evolution and workshop discussions, is provided. Firstly, stakeholders quickly realized that maximizing the built environment components (e.g., accessibility and safety design aspects) alone did not have a major impact on improving the UoS (surprising model behaviour for the participants). In comparison, strategies focusing on community interventions had larger influence on the UoS (e.g., run 2, group 2). Indeed, within the model, the use of co-design approaches had a major influence on the UoS, as it increased the maintenance demand (unintended consequence), community engagement policies, and the usability of the places. However, in practice, a small fractional change of the co-design variable indicates considerable efforts in real life, for engaging with stakeholders and for appropriate resources.

Secondly, scenarios focusing on the maintenance capacity showed, with a short delay, a slow increase of the UoS (e.g., run 2, group 1). However, this intervention depends on a substantial increase of the maintenance budget, which stakeholders reported to be difficult to achieve in practice. Yet, when the infrastructural aspects of the space are improved, we observed a lower increase of visits that tends to decrease after a certain amount of time showing that maintenance is needed to keep a suitable space condition. In fact, the decrease derives from the space condition as more people are using the space (e.g., run 2, group 2). Participants were surprised about the importance of maintenance, e.g., they found out that the focusing only on infrastructure design has a limited impact unless the quality of the space is maintained.

Thirdly, the effects can be greatly improved when attention is paid to the local community demands (through better – fit for purpose – co-design of space) or social cohesion building and community engagement activities, showing the need for combined strategies when planning interventions for increasing the UoS (e.g., run 6, group 2). Therefore, in scenarios focusing on the local community (e.g., improving the residents' knowledge of how to use the spaces and the use of co-design approaches), it is possible to observe a faster and higher improvement of the UoS in the shorter term but a decrease after certain amount of time. In this case again, the increase of the UoS indicated the demand for investing in maintenance capacity (unintended consequences). Unless the maintenance capacity is balanced with the increase of the UoS, the space condition decreased again at the end of the regeneration project (month 516), decreasing the number of visits (e.g., run 4 and 5, group 2 and run 3 group 3).

Lastly, stakeholders also focused on the social-economic aspects of the UoS and discussed interventions aimed at improving residents' availability of leisure time (e.g., run 4 group 3), represented by the variable ‘provision of local jobs and services’ in the model. This variable implies e.g., the reduction of commuting time and of the need of looking outside Thamesmead for services and work. In detail, stakeholders also mentioned a cycling route for easier commuting and some measures for reducing local crime or the deprivation rate. When the social-economic aspects are improved, the increase of visits is fast and reaches a peak after a small delay, but in order to stay at this level at the end of the regeneration project, it needs interventions around the maintenance capacity. This shows that for a fully sustained long-term effect, the traditional improvements of the infrastructural side need to be matched with the provision of new jobs and services, and other community-oriented measures related to co-design approaches and community participation (surprising model behaviour for the participants).

### 5.1 Insights from the workshop evaluation

For the purpose of self-reflection, following the ‘reflective practitioners’ (Schön, 1983) idea, the last workshop ended with a structured discussion to evaluate the workshop and gain feedback on the model. Moreover, a post-workshop evaluation survey was filled-in by the workshop participants. Recordings and survey results were reviewed to explore knowledge production and learning outcomes.

Stakeholders considered it a “*useful and fascinating session*”, with an *“intuitive and simple to use model”*. Participants agreed that the model and simulation process have given more insights into the cohesion between the variables that compose the problem, and it was helpful to understand of the opinions of the other participants. The insights from this evaluation exercise underlined the usefulness of a participatory SD modelling process for supporting discussion on a complex problem, involving solutions from multi-organizations, in a cross-sectoral context. Stakeholders also highlighted that the workshop was a suitable space to enable the sharing of values, and to prioritize and reflect together, allowing discussion on divergent viewpoints on the issue under consideration. They mentioned that the model could be used as a tool to support internal decision-making around strategy building, and a way to communicate key decisions (around natural space) to others. As one participant noted in the post-workshop evaluation survey *“I was curious about how this model could be applied to my work streams and how I could use it to influence others.*”

The stakeholders understood that the model does not predict the future, but it helps establishing deeper discussions on future-scenarios, and such discussions are valuable to reach agreement between groups and stakeholders who may have differing priorities. One participant stated they took part in the workshop because they were: *“Interested in working with experts outside of housing -* e.g. *academics to see what added value can be provided to aid decision making”.*

A few reflections on practical improvements for the model were also mentioned, including providing clarity on the data behind it. For example, one participant stated: “*It is a useful tool for discussion however with a lack of clarity about what data sits behind it then the tool cannot be as effective outside of this workshop* e.g. *with other colleagues [not involved in the participatory modelling process]*”. However, we are currently in the process of using it to develop a strategy in a Thamesmead regeneration sub-project with a core stakeholder. To conclude, the insights from the evaluation and our reflections, highlighted the model was considered useful for i. knowledge sharing and co-production within each organizations, between organizations and with local residents; ii. enabling conversations on levers and supporting debates to reach agreement; iii. structuring, managing and unpacking complexity allowing to simplify a complex set of data and thinking.

## 6 Discussion and lesson learnt

This study has contributed insights on how participatory modelling can be used to share and co-produce knowledge, between a variety of stakeholders, who hold differing perspectives based on their organisational roles and expertise. In doing so, the paper also provided an understanding of the dynamics driving the UoS in the case study area. Since the model's mechanisms and parameterisation are deeply grounded with the case study stakeholders' knowledge, the application of the scenario-based results to different areas requires further testing. However, the case study-based knowledge facilitated co-production of the information that the stakeholders needed for decision-making, aligning with the objectives of the case study.

Thanks to the modelling process, we have provided a more nuanced understanding of ‘participation’ in participatory SD modelling, bringing together different stakeholders to discuss and deliberate on the factors that influence the UoS. Specifically, we used participatory modelling, a SD model and its interface, as boundary object to bring together and engage with a varied group of stakeholders, to explore and discuss possible pathways of interventions for improving the UoS and to investigate what to prioritize, enabling participants' individual and social learning.

Our research has highlighted the value of a specific approach to bring together different stakeholders, through participatory SD, and how this leads to: the co-creation of knowledge about UoS, the development of a model in a data scarcity context, and increasing awareness and knowledge through discussion. In this section, we consider our findings in relation to existing research. First, we describe the strengths and limitations of our approach. Then we discuss potential of participatory SD to build social learning. We then consider the role of diverse knowledge types from a learning perspective. Finally, we reflect upon the potential impact of undertaking our engagement online, in relation to our findings.

This study and the model contribute to the literature in a few ways. Firstly, within a multidisciplinary and transdisciplinary setting, the model captures endogenous dynamics between the UoS, perception of safety, and community participation together with other influencing system elements (e.g., design aspects). Various factors and their relationships were captured by experts and stakeholders working on the project. Secondly, the model is grounded in the causal dynamics identified by stakeholders, and it proposed important causal pathways for increasing the UoS which could be used in future studies in different research fields. However, there are limitations with this study. The extensive participatory process was a long and resource-demanding activity for both the stakeholders and the research team; therefore, a diversity of settings was used across stakeholder groups in the process (i.e., in-person workshop, when possible, online workshop, interviews, literature review-consultation), rather than keeping to one format. Furthermore, our research combined established methods (e.g. workshops, surveys) with structured methods for self-reflection within the research process itself, as our research took a ‘learning by doing’ approach in the application, testing and refinement of the modelling process. Accompanying this was a strand of research which aims to develop an evaluation framework for participatory SD modelling. We have taken a developmental evaluation approach, similarly testing and refining evaluation processes into our study (rather than apply an established framework at the start), meaning that a range of outcomes (to people, processes, and plans) were captured. This work in ongoing, more insights will be provided on this work in forthcoming publications. Finally, limitations of our model include: (i) the model does not distinguish between the different type of activities and UoS, while different outdoor activities have a different role in the system under consideration. For instance, considering an urban natural space as a destination or as a path; future research is needed in this direction; (ii) the current version of the model does not take into consideration the costs and timeframe required to implement the suggested strategies, due to lack of data, scale issues and different responsibilities of the organizations involved; (iii) the model does not directly link the UoS to health indicators. More research to the quantification of such relationship is on-going; (iv) being the model grounded in stakeholders' knowledge, the contribution from literature on the drivers of using urban space (e.g., from urban ecology) can be improved; (v) while the participatory modelling process is replicable, the model needs to be calibrated to new areas.

With these limitations in mind, the research revealed that the participatory SD modelling approach enabled the spaces for different actors to come together, share and co-produce knowledge on the UoS. The final workshop, focused on testing scenarios and developing strategies, revealed various assumptions on the influencing factors from different stakeholders and unintended consequences. Working in small groups the stakeholders were encouraged to decide, test and reflect on potential ways to influence the system – this activity resulted in learning. Learning and knowledge production naturally go together. An important finding of our study relates to learning, which has been identified as a key feature and a normative goal of co-production (as illustrated in Audia et al., 2021's framework for equitable urban health). Within our study, the learning resulted from the collaborative or mutual development and sharing of knowledge by multiple stakeholders, aligning with what Diduck et al. (2005) terms as ‘social learning’. Rather than keeping the learning and knowledge restricted (and controlled) by the engager (in this case the research/modelling team), we adopted processes to facilitate multi-level (e.g., individuals, groups, organizations, and networks) and multi-loop (e.g., opportunities question assumptions, reframe knowledge in specific contexts) learning on the topic of UoS. Our study adds to the body of participatory modelling studies aimed to support learning – for this case study it was learning that relates to understanding use factors that influence the use of space, different ways of understanding it, and the impacts of alternative practices and plans forward (Hedelin et al., 2021). Taking a system perspective was helpful to further conceptualise the models of learning facilitated through this process. Models of multi-level, multi-loop and double-loop learning (Argyris and Schon, 1974) align with our observations from the processes, drawing on systems thinking, which can ultimately lead to altered mental models and shifts in decision-making. The modelling activities, the use of the interface and workshops within the participatory SD process gave rise to learning at different levels. For instance, the group activity on scenario testing, using the interface, in final workshop was an opportunity for deliberation and action group learning, supporting individual and collective learning. The interface and learnings from the simulation, are ‘knowledge objects’ that stakeholders can use with confidence. There is, nonetheless, an unresolved question as to whether participatory SD can move from the co-production of knowledge for learning, to the co-production of knowledge for to collective action. Corburn (2005) note the outputs of some participatory processes on policies and practices, with the resulting outputs integrating knowledge. Within our study, at this point in time, the process and model have not directly influenced any policies or plans relating to the UoS in Thamesmead; however there seems to be some influence at strategic level. This is on-going participatory activity, and more discussions are currently happening in this direction. However, the findings of this paper point towards to wider issues linked to developing capacities and capabilities of stakeholders to influence and implement changes on urban environmental issues, such as use of natural space, with an opportunity for research to examine how approaches like participatory SD can build capacities for action.

Furthermore, knowledge is a product of specific values, cultures and social relations (Argyris and Schon, 1974), thus it is important to consider the *who* is involved in the production of knowledge and learning. Within this study, efforts were made to widen the diversity of stakeholders (in terms of their professional roles/positionality) in the workshops to ensure that the SD model included various viewpoints on the UoS. However, if the goal of participatory approaches is to open up decision-making to a range of voices, then it needs to be recognized that these different voices may find it difficult to find the common links to reach consensus. In some situations, different and sometimes competing understanding of the system and discourses seemed to lead to divergence, instead of convergence, among stakeholders (e.g., see Pluchinotta et al., 2022). We argue that participatory SD modelling and the created models (both qualitative and quantitative) are useful tools to support knowledge co-production and, the developed interface, helped to highlight diversity in viewpoints but can provide a boundary object where multiple factors can come together in a ‘connected system’.

The definition of co-production, by Audia et al. (2021) that we adopted in this study, noted the complexity of the process, and that knowledge systems can be shaped not only by cooperation, by also by conflict and disagreement. Within this study, the scenario testing activity acknowledges the roles of discussion, negotiation and compromise in decision-making. A key skill of those deeply engaged in the regeneration initiatives, such as Thamesmead, is the ability to understand tensions, discuss the “why”s, seek ways of addressing them and to ultimately resolve any conflicts between individuals and groups, notes that there is a general unwillingness of many partnership initiatives to engage with conflict, resulting in a failure to accept and work with difference. Our study, taking a systems approach, draws attention to the multitude of perspectives, interpretations and agendas that exist. Differences in values and understandings were evident. For example, within the ‘strategy development and learning’ workshop, for several stakeholders involved, the maintenance of space and the accessibility were of importance, however for others there was a desire to focus on community activities and the social aspect of the UoS. The participatory approach presented in this paper enabled a process of cooperation and collaboration, either where agreement was reached or an understanding of why a certain option was decided.

In conclusion, this study has developed a participatory SD modelling process and the creation of a simulation model as an approach to build learning and co-produce novel knowledge on the use of natural space, whilst taking a holistic approach to investigate the factors influencing use. The study outlines a novel tool to identify ways of increasing the value of urban natural space focusing on urban areas undergoing physical and social transformation.

## CRediT authorship contribution statement

**Irene Pluchinotta:** Conceptualization, Formal analysis, Investigation, Methodology, Project administration, Software, Visualization, Writing – original draft, Writing – review & editing. **Ke Zhou:** Writing – original draft, Writing – review & editing, Data curation, Formal analysis, Investigation, Methodology. **Gemma Moore:** Conceptualization, Methodology, Writing – original draft, Writing – review & editing. **Giuseppe Salvia:** Writing – review & editing. **Kristine Belesova:** Data curation, Writing – review & editing. **Nahid Mohajeri:** Writing – review & editing. **Joanna Hale:** Writing – review & editing. **Michael Davies:** Funding acquisition. **Nici Zimmermann:** Methodology, Supervision, Writing – review & editing.

## Declaration of competing interest

The authors declare that they have no known competing financial interests or personal relationships that could have appeared to influence the work reported in this paper.

## Data Availability

Data will be made available on request.

## Footnotes

1 https://projectcussh.org/.

2 https://www.camelliawater.org/.

3 The model interface described in this paper is available here: https://exchange.iseesystems.com/public/ucl/thamesmead-use-of-natural-space-model. The authors worked on a second version of this model after submitting the present manuscript. The new version explores the effects on health of the use of natural space: https://exchange.iseesystems.com/public/ucl/thamesmead-use-of-natural-space--health.

4 Stella Architect - isee systems, https://www.iseesystems.com/store/products/stella-architect.aspx.
