# Supplementary material for: Co-producing knowledge on the use of urban natural space: Participatory system dynamics modelling to understand a complex urban system
Source: J Environ Manage. 2024 Feb 27;353:120110. doi: 10.1016/j.jenvman.2024.120110 (PMC11129192; doi:10.1016/j.jenvman.2024.120110)
Supplement: Multimedia component 1 [file mmc1.docx]

**Co-producing knowledge on the use of urban natural space: participatory System Dynamics modelling to understand a complex urban system**

Irene Pluchinotta*, Ke Zhou, Gemma Moore, Giuseppe Salvia, Kristine Belesova, Nahid Mohajeri^,^ Joanna Hale, Michael Davies, Nici Zimmermann

Contact: [i.pluchinotta@ucl.ac.uk](mailto:i.pluchinotta@ucl.ac.uk)

**Supplementary Material**

**Appendix I**

This section includes additional details related to the workshops developed during the participatory System Dynamics (SD) modelling process described in Section 3 and Table 1 of the manuscript. Following a chronological order of the stakeholder activities, the agenda of the ‘strategy development and learning’ workshop (described in Section 5 of the manuscript), is at the end of this Appendix.

The focus of the simulation model was the Use of urban natural Space (UoS); however, the workshop structure is easily generalizable for other models.

*Model quantification workshop*

Supplementary Materials Table 1. Agenda of the participatory model quantification workshop

| **Participatory model quantification workshop (90 min)** | |
| --- | --- |
| *Aims:* to present and validate the structure of the SD simulation model, to help quantify specific sections of the model  *Location:* online (Microsoft Teams) | |
| **Time** | **Activity** |
| 5 min | Welcome and workshop objectives |
| 15 min | Presentation of a simplified version of the SD model using a CLD when needed, including model assumptions and calibration process |
| 25 min | Structured discussion for validating the model structure |
| 5 min | Introduction of the drawing BOT graphs activity |
| 25 min | BOT activity for a selection of variables. Stakeholders are divided in groups |
| 10 min | Presentation of the BOT graphs created by the groups |
| 5 min | Next steps and closing |

*Reference model validation workshop*

| **Reference model validation workshop (60 min)** | | |
| --- | --- | --- |
| *Aims:* to present the analysis of the historical dataset used as reference model  *Location:* online (Microsoft Teams) | | |
| **Time** | **Activity** | **Description** |
| 10 min | Welcome and workshop objectives | - |
| 10 min | Introduction to the historical dataset (MENE, Natural England) and report on the analysis | - |
| 25 min | Structured discussion for confirming the reference model relevance to the model. | Stakeholders answers specific questions around the dataset and the case study area (e.g., Has anything relevant happened in 2018 in relation to the natural spaces in Thamesmead?​). Afterwards, the group discussed their interpretation of the analysis especially in relation to the urban regeneration plan and other stakeholders’ ambitions |
| 15 min | Next steps and closing | - |

*Scenarios building workshop*

| **Scenarios building workshop (60 min)** | | |
| --- | --- | --- |
| *Aims:* to present the analysis of the historical dataset used as reference model  *Location:* online (Microsoft Teams) | | |
| **Time** | **Activity** | **Description** |
| 5 min | Welcome and workshop objectives | - |
| 5 min | Introduction to the scenarios building methodology | Adapted from Pluchinotta et al., 2019^[[1]](#footnote-1)^ |
| 15 min | Idea generation on “how to improve the use of space in Thamesmead” – session 1 | Gathering initial thoughts on the problem under consideration. Participants are asked to write one idea on each post-it and report back to the group. The facilitator grouped the post-it in thematic clusters. After each participant has had a chance to share once, the facilitator opens the floor to participants to offer also more options |
| 5 min | Knowledge expansion and CLD presentation. | Bringing to the discussion the key aspects of the model CLD to help the participants identifying possible solutions focusing on ALL the areas of the model. |
| 25 min | Idea generation – session 2 | Participants are asked to write a post-it for each possible actions in one or more of the CLD areas. Then similar to session 1. |
| 5 min | Closing |  |

*Strategy development and learning*

| **Strategy development and learning workshop agenda (3h)** | | |
| --- | --- | --- |
| *Aims:* (i) to present the SD simulation model; (ii) to allow the stakeholders to explore and interact with the model via the model interface; (iii) to discuss the key elements influencing the dynamics of the issue under consideration to inform decision-making; (iv) to discuss how to use the model and plan the next steps in a participatory way  *Location:* online (Microsoft Teams) | | |
| **Time** | **Activity** | **Description** |
| 10 min | Welcome, workshop objectives and summary of the past activities and modelling process | - |
| 10 min | Model description | The model description included e.g., the dynamics of the UoS in Thamesmead, causal relationships and independencies, model assumptions |
| 10 min | Selection of the model variables that each participants consider important for improving the UoS | This is an individual activity supported by an online board (e.g., Miro https://miro.com), aimed at capturing the preliminary thoughts on the most influential elements of the system. This activity was done without interacting with the model interface. |
| 10 min | Model interface presentation, and activity explanation | - |
| 60 min | Strategy Development: Play and Test | This is a group activity; breakout rooms on Microsoft Teams were used. Each group was supported by a facilitator and note taker. The objective was, for the group, to jointly agree on the first set of 3 variables to change, discussing the intensity of change and the rationale behind it. The outcome of the previous activity could be used as starting point. Each scenario agreed named and then ran via the interface and discussed by the group. Screenshots of each scenario are stored on the online board by the notetaker. This process is repeated until the group is satisfied with the outcome or the time is up. |
| 30 min | Plenary discussion after the activity Strategy Development: Play and Test | Each group presented what they considered the best strategy to trigger a plenary discussion |
| 5 min | Modellers’ scenarios presentation | - |
| 30 min | Discussion on the model application and future steps | - |
| 10 min | Evaluation survey | - |
| 5 min | Closing | - |

**Appendix II**

This section includes additional details related to the SD simulation model on the Use of urban natural Space (UoS) described in Section 4 of the manuscript. Dmnl = dimensionless

The model interface described in this paper is available here: <https://exchange.iseesystems.com/public/ucl/thamesmead-use-of-natural-space-model> . The authors worked on a second version of this model after submitting the present manuscript. The new version explores the effects on health of the use of natural space: <https://exchange.iseesystems.com/public/ucl/thamesmead-use-of-natural-space--health> .

Overview of the Use of urban natural Space (UoS) System Dynamics (SD) simulation model.

| **Variables** | **Equations** | **Units** |
| --- | --- | --- |
| Adjusted_crime_level | Average_London_crime_level*Effect_of_UoS_on_crime | dmnl |
| Average_London_crime_level | 4.5 | dmnl |
| Average_London_deprivation_level | 5 | dmnl |
| Average_ratio_of_UoS | SAFEDIV("Weekly_visits_per_person_(UoS)", Maximum_number_of_visits_per_week_per_people) | dmnl |
| Baseline_attitude_towards_environment | 0.5 | dmnl |
| Baseline_biodiversity | 0.3 | dmnl |
| "Baseline_co-design" | 0 | dmnl |
| Baseline_maintenance_capacity | 0.2 | dmnl |
| Baseline_providing_local_jobs_and_services | 0.5 | dmnl |
| Baseline_resident_knowledge_about_UoS | 0.5 | dmnl |
| Baseline_safety_design | 0.15 | dmnl |
| Baseline_space_accessibility | 0.35 | dmnl |
| Behaviour_change_time | 36 | Month |
| Change_of_safety_perception | SAFEDIV((Indicated_safety_perception-Residents'_perceived_safety_level), Perception_change_time) | Per month |
| Change_of_UoS | (Indicated_level_of_UoS-"Weekly_visits_per_person_(UoS)")/Behaviour_change_time | Visits/ week/people/month |
| Community_participation | Effect_of_space_condition_on_community_participation*DELAY3(Residents_awareness_of_UoS_opportunities*TOTAL_intangible_effect_on_community_participation, Behaviour_change_time) | dmnl |
| Effect_of_accessibility_on_usability | GRAPH((1-Regeneration_programme_switch)* Baseline_space_accessibility +Regeneration_programme_switch*REGENERATION_space_accessibility) Points: (0.000, 0.000), (0.100, 0.33583091167), (0.200, 0.560945103841), (0.300, 0.7118436595), (0.400, 0.812993986277), (0.500, 0.880797077978), (0.600, 0.926246849528), (0.700, 0.956712742486), (0.800, 0.977134641257), (0.900, 0.99082384938), (1.000, 1.000) | dmnl |
| "Effect_of_co-design_approach_on_change_of_residents_awareness_of_space_and_participation" | GRAPH("Effect_of_co-design_approach_on_usability") Points: (0.000, 1.00334642546), (0.100, 1.00899310498), (0.200, 1.02371293659), (0.300, 1.05960146101), (0.400, 1.13447071069), (0.500, 1.2500), (0.600, 1.36552928932), (0.700, 1.44039853899), (0.800, 1.47628706341), (0.900, 1.49100689502), (1.000, 1.49665357454) | dmnl |
| "Effect_of_co-design_approach_on_maintenance_demand_and_costs" | GRAPH("Use_of_co-design_approach") Points: (0.000, 1.00669285092), (0.100, 1.01798620996), (0.200, 1.04742587318), (0.300, 1.11920292202), (0.400, 1.26894142137), (0.500, 1.500), (0.600, 1.73105857863), (0.700, 1.88079707798), (0.800, 1.95257412682), (0.900, 1.98201379004), (1.000, 1.99330714908) | dmnl |
| "Effect_of_co-design_approach_on_usability" | "Use_of_co-design_approach" | dmnl |
| Effect_of_deprivation_and_crime_on_perception | GRAPH((Average_London_deprivation_level+Adjusted_crime_level)/2) Points: (0.00, 0.00334642546214), (1.00, 0.00899310498105), (2.00, 0.0237129365888), (3.00, 0.0596014610111), (4.00, 0.134470710685), (5.00, 0.250), (6.00, 0.365529289315), (7.00, 0.440398538989), (8.00, 0.476287063411), (9.00, 0.491006895019), (10.00, 0.496653574538) | dmnl |
| Effect_of_deprivation_on_leisure_time | GRAPH(Average_London_deprivation_level) Points: (0.00, 0.998707650224), (1.00, 0.995131068756), (2.00, 0.981836308972), (3.00, 0.934624666574), (4.00, 0.790840634787), (5.00, 0.750), (6.00, 0.209159365213), (7.00, 0.0653753334256), (8.00, 0.0181636910284), (9.00, 0.00486893124376), (10.00, 0.00129234977629) | dmnl |
| Effect_of_design_aspects_on_perception_of_safety | GRAPH("Safety-design_of_the_space") Points: (0.000, 0.000), (0.100, 0.33583091167), (0.200, 0.560945103841), (0.300, 0.7118436595), (0.400, 0.812993986277), (0.500, 0.880797077978), (0.600, 0.926246849528), (0.700, 0.956712742486), (0.800, 0.977134641257), (0.900, 0.99082384938), (1.000, 1.000) | dmnl |
| Effect_of_leisure_time_on_UoS | GRAPH(Leisure_time_for_UoS) Points: (0.0, 0.00200785527729), (48.0, 0.00539586298863), (96.0, 0.0142277619533), (144.0, 0.0357608766066), (192.0, 0.080682426411), (240.0, 0.1500), (288.0, 0.219317573589), (336.0, 0.264239123393), (384.0, 0.285772238047), (432.0, 0.294604137011), (480.0, 0.297992144723) | dmnl |
| Effect_of_residents_knowledge_on_awareness_of_opportunities | Resident_knowledge_about_UoS | dmnl |
| Effect_of_soft_interventions_on_residents'_awareness | (Residents'_environmental_attitude+Effect_of_residents_knowledge_on_awareness_of_opportunities+"Effect_of_co-design_approach_on_change_of_residents_awareness_of_space_and_participation")/3 | dmnl |
| Effect_of_space_condition_on_community_participation | GRAPH(Urban_Natural_Space_Condition) Points: (0.000, 1.00669285092), (0.100, 1.01798620996), (0.200, 1.04742587318), (0.300, 1.11920292202), (0.400, 1.26894142137), (0.500, 1.500), (0.600, 1.73105857863), (0.700, 1.88079707798), (0.800, 1.95257412682), (0.900, 1.98201379004), (1.000, 1.99330714908) | dmnl |
| Effect_of_space_condition_on_usability_and_perception | GRAPH(Urban_Natural_Space_Condition) Points: (0.000, 0.014), (0.100, 0.0179862099621), (0.200, 0.0474258731776), (0.300, 0.119202922022), (0.400, 0.26894142137), (0.500, 0.500), (0.600, 0.73105857863), (0.700, 0.880797077978), (0.800, 0.952574126822), (0.900, 0.982013790038), (1.000, 0.993307149076) | dmnl |
| Effect_of_space_use_on_decay_time | GRAPH(Average_ratio_of_UoS) Points: (0.000, 2.000), (0.100, 1.99082384938), (0.200, 1.97713464126), (0.300, 1.95671274249), (0.400, 1.92624684953), (0.500, 1.88079707798), (0.600, 1.81299398628), (0.700, 1.7118436595), (0.800, 1.56094510384), (0.900, 1.33583091167), (1.000, 1.000) | dmnl |
| Effect_of_UoS_on_crime | GRAPH("Weekly_visits_per_person_(UoS)") Points: (0.000, 1.87982669965), (0.100, 1.80049902176), (0.200, 1.67778210085), (0.300, 1.50052021119), (0.400, 1.26827118202), (0.500, 1.000), (0.600, 0.731728817978), (0.700, 0.49947978881), (0.800, 0.322217899153), (0.900, 0.199500978239), (1.000, 0.120173300348) | dmnl |
| Effect_of_UoS_on_participation | GRAPH(Average_ratio_of_UoS) Points: (0.000, 0.0000), (0.100, 0.167915455835), (0.200, 0.280472551921), (0.300, 0.35592182975), (0.400, 0.406496993138), (0.500, 0.440398538989), (0.600, 0.463123424764), (0.700, 0.478356371243), (0.800, 0.488567320628), (0.900, 0.49541192469), (1.000, 0.5000) | dmnl |
| Effect_of_UoS_on_perception | GRAPH(Average_ratio_of_UoS) Points: (0.000, 0.00669285092428), (0.100, 0.0179862099621), (0.200, 0.0474258731776), (0.300, 0.119202922022), (0.400, 0.26894142137), (0.500, 0.500), (0.600, 0.73105857863), (0.700, 0.880797077978), (0.800, 0.952574126822), (0.900, 0.982013790038), (1.000, 0.993307149076) | dmnl |
| Effect_of_usability_on_perception | GRAPH(Usability_of_urban_natural_spaces) Points: (0.000, 0.00669285092428), (0.100, 0.0179862099621), (0.200, 0.0474258731776), (0.300, 0.119202922022), (0.400, 0.26894142137), (0.500, 0.500), (0.600, 0.73105857863), (0.700, 0.880797077978), (0.800, 0.952574126822), (0.900, 0.982013790038), (1.000, 0.993307149076) | dmnl |
| Effect_of_usability_on_residents'_awareness | GRAPH(Usability_of_urban_natural_spaces) Points: (0.000, 1.00669285092), (0.100, 1.01798620996), (0.200, 1.04742587318), (0.300, 1.11920292202), (0.400, 1.26894142137), (0.500, 1.500), (0.600, 1.73105857863), (0.700, 1.88079707798), (0.800, 1.95257412682), (0.900, 1.98201379004), (1.000, 1.99330714908) | dmnl |
| Effectiveness_regeneration_on_improving_space | 1 | dmnl |
| Effects_of_built_and_natural_environment_influence_on_UoS | GRAPH((Usability_of_urban_natural_spaces+Biodiversity)/2) Points: (0.000, 0.000552778636924), (0.100, 0.00247262315663), (0.200, 0.0109869426306), (0.300, 0.0474258731776), (0.400, 0.182425523806), (0.500, 0.500), (0.600, 0.817574476194), (0.700, 0.952574126822), (0.800, 0.989013057369), (0.900, 0.997527376843), (1.000, 0.999447221363) | dmnl |
| Effects_of_perception_and_awareness_on_UoS | GRAPH((Residents'_perceived_safety_level+Residents_awareness_of_UoS_opportunities+Community_participation)/3) Points: (0.000, 0.000746028833837), (0.100, 0.00314121328483), (0.200, 0.0131253183371), (0.300, 0.0531511363981), (0.400, 0.191545348561), (0.500, 0.500), (0.600, 0.808454651439), (0.700, 0.946848863602), (0.800, 0.986874681663), (0.900, 0.996858786715), (1.000, 0.999253971166) | dmnl |
| Fastest_space_decay_time | 84 | Month |
| Gap_of_space_condition | (Goal_space_condition-Urban_Natural_Space_Condition) | dmnl |
| Goal_space_condition | 0.7 | dmnl |
| Indicated_level_of_UoS | TOTAL_effects_on_UoS*Maximum_number_of_visits_per_week_per_people | Visits/ week/people |
| Indicated_safety_perception | TOTAL_effects_on_perception*Normal_safety_perception | dmnl |
| Leisure_time_for_UoS | Normal_weekly_leisure_time_for_UoS*Effect_of_deprivation_on_leisure_time*((Regeneration_programme_switch*REGENERATION_providing_local_jobs_and_services)+(1-Regeneration_programme_switch)*Baseline_providing_local_jobs_and_services) | Minutes/week/people |
| Maintenance_capacity | Regeneration_programme_switch*REGENERATION_maintenance_capacity+(1-Regeneration_programme_switch)*Baseline_maintenance_capacity | dmnl |
| Maintenance_demand_per_month | Space_condition_decreasing*"Effect_of_co-design_approach_on_maintenance_demand_and_costs" | 1/month |
| Maximum_maintenance_rate_per_month | Maintenance_capacity//Space_decay_time | 1/month |
| Maximum_number_of_visits_per_week_per_people | 5 | Visits/ week/people |
| Normal_residents_awareness | 0.3 | dmnl |
| Normal_safety_perception | 1 | dmnl |
| Normal_space_usability | 1 | dmnl |
| Normal_weekly_leisure_time_for_UoS | 120 | Minutes/week/people |
| Perception_change_time | 36 | Month |
| REGENERATION_attitude_towards_environment | 0.5 | dmnl |
| "REGENERATION_co-design" | 0 | dmnl |
| REGENERATION_maintenance_capacity | 0.2 | dmnl |
| "REGENERATION_policy_switch_1=RegenerationOn" | 1 | dmnl |
| Regeneration_programme_switch | IF TIME>Regeneration_project_starting_time THEN "REGENERATION_policy_switch_1=RegenerationOn" ELSE 0 | dmnl |
| Regeneration_project_starting_time | 216 | Month |
| Regeneration_project_stop_time | Regeneration_project_time+Regeneration_project_starting_time | Month |
| Regeneration_project_time | 300 | Month |
| REGENERATION_providing_local_jobs_and_services | 0.5 | dmnl |
| REGENERATION_resident_knowledge_about_UoS | 0.5 | dmnl |
| "REGENERATION_safety-design_aspects" | 0.15 | dmnl |
| REGENERATION_space_accessibility | 0.35 | dmnl |
| Resident_knowledge_about_UoS | Regeneration_programme_switch*REGENERATION_resident_knowledge_about_UoS+(1-Regeneration_programme_switch)*Baseline_resident_knowledge_about_UoS | dmnl |
| Residents_awareness_of_UoS_opportunities | SMTH3 ((Normal_residents_awareness*Effect_of_usability_on_residents'_awareness*Effect_of_soft_interventions_on_residents'_awareness), Perception_change_time) | dmnl |
| Residents'_environmental_attitude | Regeneration_programme_switch*REGENERATION_attitude_towards_environment+(1-Regeneration_programme_switch)*Baseline_attitude_towards_environment | dmnl |
| Residents'_perceived_safety_level(t) | Residents'_perceived_safety_level(t - dt) + (Change_of_safety_perception) * dt | dmnl |
| "Safety-design_of_the_space" | Baseline_safety_design*(1-Regeneration_programme_switch)+Regeneration_programme_switch*"REGENERATION_safety-design_aspects" | dmnl |
| Space_condition_being_improved_by_maintenance | MIN(Maximum_maintenance_rate_per_month, Maintenance_demand_per_month) | 1/month |
| Space_condition_being_improved_by_regeneration | MAX(IF TIME > Regeneration_project_stop_time THEN 0 ELSE (STEP(Gap_of_space_condition, Regeneration_project_starting_time)-STEP(Gap_of_space_condition, Regeneration_project_stop_time))*Regeneration_programme_switch//Regeneration_project_time*Effectiveness_regeneration_on_improving_space, 0) | 1/month |
| Space_condition_decreasing | Urban_Natural_Space_Condition//Space_decay_time | 1/month |
| Space_decay_time | Fastest_space_decay_time * Effect_of_space_use_on_decay_time | Month |
| "Starting_month_of_co-design" | 252 | Month |
| TOTAL_effect_on_usability | ("Effect_of_co-design_approach_on_usability"^(1-"Weight_of_use_of_co-design_approaches_on_usability")+Effect_of_accessibility_on_usability^(1-Weight_of_accessibility_on_usability)+Effect_of_space_condition_on_usability_and_perception^(1-Weight_of_space_condition_on_usability))/3 | dmnl |
| TOTAL_effects_on_perception | (Effect_of_design_aspects_on_perception_of_safety^(1-"Weight_of_safety-design_aspects_on_perception")+Effect_of_usability_on_perception^(1-Weight_of_usability_on_perception)+Effect_of_UoS_on_perception^(1-Weight_of_UoS_on_perception)+Effect_of_space_condition_on_usability_and_perception^(1-Weight_of_space_condition_on_perception)-Effect_of_deprivation_and_crime_on_perception^(1-Weight_of_deprivation_and_crime_on_perception))/5 | dmnl |
| TOTAL_effects_on_UoS | (Effects_of_built_and_natural_environment_influence_on_UoS^(1-"Weight_of_built/natural_environment_on_UoS")+Effects_of_perception_and_awareness_on_UoS^(1-Weight_of_perception_and_awareness_on_UoS)+Effect_of_leisure_time_on_UoS^(1-Weight_of_leisure_time_on_UoS))/3 | dmnl |
| TOTAL_intangible_effect_on_community_participation | (Effect_of_soft_interventions_on_residents'_awareness^(1-Weight_of_soft_interventions_on_community_participation)+"Effect_of_co-design_approach_on_change_of_residents_awareness_of_space_and_participation"^(1-"Weight_of_co-design_approaches_on_community_participation")+Effect_of_UoS_on_participation^(1-Weight_of_UoS_influence_on_community_participation))/3 | dmnl |
| Urban_Natural_Space_Condition(t) | Urban_Natural_Space_Condition(t - dt) + (Space_condition_being_improved_by_maintenance + Space_condition_being_improved_by_regeneration - Space_condition_decreasing) * dt | dmnl |
| Usability_of_urban_natural_spaces | TOTAL_effect_on_usability*Normal_space_usability | dmnl |
| "Use_of_co-design_approach" | IF TIME<"Starting_month_of_co-design" THEN "Baseline_co-design" ELSE "REGENERATION_co-design"*Regeneration_programme_switch | dmnl |
| Weekly_visits_from_MENE_data | 0.6326522 |  |
| "Weekly_visits_per_person_(UoS)"(t) | "Weekly_visits_per_person_(UoS)"(t - dt) + (Change_of_UoS) * dt | Visits/ week/people |
| Weight_of_accessibility_on_usability | 0.4 | dmnl |
| "Weight_of_built/natural_environment_on_UoS" | 0.2 | dmnl |
| "Weight_of_co-design_approaches_on_community_participation" | 0.4 | dmnl |
| Weight_of_deprivation_and_crime_on_perception | 0.3 | dmnl |
| Weight_of_leisure_time_on_UoS | 0.4 | dmnl |
| Weight_of_perception_and_awareness_on_UoS | 0.4 | dmnl |
| "Weight_of_safety-design_aspects_on_perception" | 0.2 | dmnl |
| Weight_of_soft_interventions_on_community_participation | 0.4 | dmnl |
| Weight_of_space_condition_on_perception | 0.2 | dmnl |
| Weight_of_space_condition_on_usability | 0.4 | dmnl |
| Weight_of_UoS_influence_on_community_participation | 0.2 | dmnl |
| Weight_of_UoS_on_perception | 0.2 | dmnl |
| Weight_of_usability_on_perception | 0.1 | dmnl |
| "Weight_of_use_of_co-design_approaches_on_usability" | 0.2 | dmnl |
| Biodiversity_output: |  |  |
| Biodiversity | Baseline_biodiversity*TOTAL_Effect_on_biodiversity | dmnl |
| Effect_of_active_travel_on_biodiversity | Active_travel/Active_travel | dmnl |
| Effect_of_maintenance_on_biodiversity | GRAPH(Space_condition_being_improved_by_maintenance) Points: (0.000, 0.00669285092428), (0.100, 0.0179862099621), (0.200, 0.0474258731776), (0.300, 0.119202922022), (0.400, 0.26894142137), (0.500, 0.500), (0.600, 0.73105857863), (0.700, 0.880797077978), (0.800, 0.952574126822), (0.900, 0.982013790038), (1.000, 0.993307149076) | dmnl |
| Effect_of_UoS_spaces_on_biodiversity | GRAPH(Average_ratio_of_UoS) Points: (0.000, 0.00334642546214), (0.100, 0.00899310498105), (0.200, 0.0237129365888), (0.300, 0.0596014610111), (0.400, 0.134470710685), (0.500, 0.2500), (0.600, 0.365529289315), (0.700, 0.440398538989), (0.800, 0.476287063411), (0.900, 0.491006895019), (1.000, 0.496653574538) | dmnl |
| TOTAL_Effect_on_biodiversity | (Effect_of_maintenance_on_biodiversity^(1-Weight_of_maintenance_on_biodiversity)+Effect_of_active_travel_on_biodiversity^(1-Weight_of_active_travel_on_biodiversity)-Effect_of_UoS_spaces_on_biodiversity^(1-Weight_of_UoS_on_biodiversity))/3 | dmnl |
| Weight_of_active_travel_on_biodiversity | 0.3 | dmnl |
| Weight_of_maintenance_on_biodiversity | 0.2 | dmnl |
| Weight_of_UoS_on_biodiversity | 0.5 | dmnl |
|  |  |  |
| Initial_numbers: |  |  |
| Initial_active_travel_level | 0.2 | dmnl |
| Initial_perceived_safety | 0.5 | dmnl |
| Initial_space_condition | 0.543 | dmnl |
| Initial_UoS | 2 | Visits/ week/people |

**Appendix III**

This table outlines the sources of all variables of the Use of urban natural Space (UoS) model described in Section 4 of the manuscript.

| **Literature** | **Dataset** | **Academic experts** | **Stakeholders** | **Auxiliary variables** |
| --- | --- | --- | --- | --- |
| Normal weekly leisure time for UoS  Effect of leisure time on UoS  Residents' perceived safety | Average London crime level  Average London deprivation level  Weekly visits from MENE data | Effect of accessibility on usability  Effect of co-design approach on change of residents’ awareness of space and participation  Effect of co-design approach on maintenance demand and costs  Effect of co-design approach on usability  Effect of deprivation and crime on perception  Effect of deprivation on leisure time  Effect of design aspects on perception of safety  Effect of residents’ knowledge on awareness of opportunities  Effect of soft interventions on residents' awareness  Effect of space condition on community participation  Effect of space condition on usability and perception  Effect of space use on decay time  Effect of UoS on crime  Effect of UoS on participation  Effect of UoS on perception  Effect of usability on perception  Effect of usability on residents' awareness  Effects of built and natural environment influence on UoS  Effects of perception and awareness on UoS  Leisure time for UoS  Perception change time  Fastest space decay time  Effect of active travel on biodiversity  Effect of maintenance on biodiversity  Effect of UoS spaces on biodiversity | Baseline attitude towards environment  Baseline biodiversity  Baseline co-design  Baseline maintenance capacity  Baseline providing local jobs and services  Baseline resident knowledge about UoS  Baseline safety design  Baseline space accessibility  Behaviour change time  Initial active travel level  Initial perceived safety  Initial space condition  Initial UoS  Goal space condition  Normal residents’ awareness  Normal safety perception  Normal space usability  REGENERATION attitude towards environment  REGENERATION co-design  REGENERATION maintenance capacity  REGENERATION providing local jobs and services  REGENERATION residents’ knowledge about UoS  REGENERATION safety-design aspects  REGENERATION space accessibility  Regeneration project starting time  Regeneration project stop time  Regeneration project time  · | Adjusted crime level  Average ratio of UoS  Change of safety perception  Change of UoS  Community participation  Effectiveness regeneration on improving space  Gap of space condition  Indicated level of UoS  Indicated safety perception  Regeneration programme switch  Resident knowledge about UoS  Residents’ awareness of UoS opportunities  Residents' environmental attitude  Safety-design of the space  Space condition being improved by maintenance  Space condition being improved by regeneration  Space condition decreasing  Space decay time  Starting month of co-design  TOTAL effect on usability  TOTAL effects on perception  TOTAL effects on UoS  TOTAL intangible effect on community participation  Urban Natural Space Condition  Usability of urban natural spaces  Use of co-design approach  Weekly visits per person  Maintenance capacity  Biodiversity  TOTAL Effect on biodiversity  Maintenance demand per month  Maximum maintenance rate per month  Maximum number of visits per week per people |
|  |  |  |  |  |

1. Pluchinotta et al. (2019), ‘Design Theory for Generating Alternatives in Public Decision Making Processes’, Group Decision and Negotiation, 28, 341–375 <https://doi.org/10.1007/s10726-018-09610-5> [↑](#footnote-ref-1)
